# Challenges and Limitations of Human Oversight in Ethical Artificial Intelligence Implementation in Health Care: Balancing Digital Literacy and Professional Strain

**DOI:** 10.1016/j.mcpdig.2024.08.004

**Published:** 2024-09-07

**Authors:** Roanne van Voorst

**Affiliations:** Anthropology Department, University of Amsterdam, Amsterdam, Netherlands

“Does a weatherman understand how the algorithm predicting the weather works? Will that weatherman be held responsible if the algorithm turns out to be wrong? Why should this be different for doctors?” Three rhetorical questions posed by a professor of ethical artificial intelligence (AI) who is concerned about the regulation surrounding human-nonhuman (algorithmic) decisions in health care.

The professor participated in roundtable discussions with 121 doctors and nurses who work with algorithms in their daily work, along with 35 ethicists and software engineers. The discussions were part of an ongoing, international, 5-year anthropological research project called Health-AI, funded by the European Research Committee (grant no. 101077251).^1^ It focuses on the digitization of health care and the ethical dilemmas that arise around human-nonhuman collaboration. The research involves a series of focus group interviews and roundtables, as well as ethnographic fieldwork in hospitals in the Netherlands, China, Norway, Estonia, Israel, and the United Arab Emirates.

Time and time again, participants raised an issue that is currently receiving inadequate attention in debates about ethical AI in health care. Namely, the problem that the increase of algorithmic technology is accompanied by an unrealistic expectation for professional caretakers to fully understand it, so they can serve as effective human oversighters in human-nonhuman (algorithmic) decision making. The oversighting role of humans is 1 necessary condition for labeling AI as “ethical,” where the idea is that the algorithm provides a calculation or advice to the human physician and it is then up to that human physician to either follow or deviate from that advice. This idea is naïve for 4 reasons.

### From Individuation to Hybrid Decision-Makers

First, the concept of a human autonomously making decisions separate from a computer is outdated.^2^^,^^3^ An increasing group of scholars from various disciplines believe that “individuation”—the idea that a human makes decisions independently of other elements in the world around them—is incorrect.^4^ Technology continually influences us, especially algorithms with which we interact,^5^ for example, because algorithms operate as black boxes that obstruct human oversight^6^, ^7^, ^8^, ^9^ or because the constant algorithmic modification in machine learning impedes human oversight.^10^^,^^11^ There is a growing body of research that suggests humans have a tendency to overtrust computer systems.^12^, ^13^, ^14^ This even goes for relatively simple algorithms, such as systems that are essentially nothing more than a digital protocol. For many people, algorithms carry an aura of objectivity, resulting in a “where there’s smoke, there’s fire” effect. This has been seen in studies among judges^15^ and police officers^15^, ^16^, ^17^ as well as recent events involving tax advisors.^18^ In other cases, the opposite seems to occur: there are known instances of “AI fatigue” among attending physicians who have encountered false-positive results from the system so frequently that they consciously ignore alarms.^19^ Whether a professional caretaker is alarmed by the result of an algorithm or consciously ignores it because they get annoyed by it, in both cases, the computer influences the decision and behavior of the physician. The type of influence, when, and on which physician—there is still little clarity on these matters in science, and until then, it is important to be aware of this gap in our understanding.

### Computational Experts or Medical Experts?

Second, AI produces outcomes in a manner that is not easily understandable or verifiable for most professional caretakers. This applies not only to complex AI owing to their much-discussed black boxes but also to what has been proposed as a solution to this problem of working with opaque systems, namely, the increasingly loud call of clinicians, lawmakers, and researchers for explainable AI models for high-risk areas, including health care.^20^^,^^21^ In an alarming article in *The Lancet*, Ghassemi et al^22^ argue that current explainability methods cannot provide a clear and reliable explanation for each individual decision made by the AI system. Although current explainability techniques can provide general information about how the AI system works, they are not reliable or detailed enough to explain individual decisions. The authors thus caution against having explainability be a requirement for clinically deployed models and advocate for rigorous internal and external validation of AI models as a more direct means of achieving the goals often associated with explainability. This commentary aligns with that view and adds that this goes also more generally, for the idea that professional caretakers can and should be the responsible supervisors of AI.

Doctors and nurses have been trained for years in medical processes, not computational processes—this is a difference in experience, knowledge, and perhaps even talent that, with some exceptions, as I publish elsewhere,^23^ cannot be bridged with just a few short courses on explainable or transparent AI. However, although professional caretakers may only have a superficial understanding of the algorithm they work with on a daily basis, they are still expected to check it for incorrect outcomes in the daily practice of their work. With this expectation, there looms a risk that in the years to come, the responsibility could increasingly shift from the designers and procurers of technology to the users—the professional caretakers. If the algorithm makes a mistake, it is the clinician who is held responsible: they were supposed to evaluate whether the AI system is working accurately. Already, research shows that it is often unclear who is responsible, or even accountable, for errors made by AI.^24^^,^^25^

A similar problem exists when it comes to the co-creation of ethical AI design, where professional caretakers are expected to collaborate with coders and AI developers. This is certainly an understandable and even necessary desire for an algorithm to work in a way that aligns with the practices of doctors. However, in practice, research shows that during co-creative processes, doctors and programmers not only use different discourse but also have different ideas about what, for example, constitutes an error or what a well-functioning algorithm is.^26^^,^^27^ This means that although co-creation is definitely preferable to isolated working and testing of technology, expectations for a fully trustworthy outcome should not be set too high.

For a human to be effective as an overseer, it is necessary for them to undergo further training or deepen their understanding of how AI works so that they become, as it is often said nowadays, “digitally literate.” This is not a 1-time course. Given the rapid development of AI, doctors who want to stay current actually need to constantly educate themselves on the dynamics of algorithms—and this is nearly impossible in a context where many doctors lack the time and resources to provide the best care to patients. The result, in the words of a professor in cardiology and clinician during an interview at a hospital in the Netherlands, is “trainings are being attended en masse to check off the checkbox, while our minds are actually elsewhere—already with the next patient in the waiting room.”

### Time Pressure

Another issue is that with the advent of AI, there is often an expectation that AI will make health care more time efficient. In other words, now that professional caretakers are receiving assistance from AI, health care tasks are expected to be completed more quickly. Research shows that this narrative is widespread, both in health care and in the technological industry that increasingly supports health care, often influenced by policymakers.^28^ Research also indicates that these expectations of working more efficiently can actually lead to a lack of time for professional caretakers to oversee the outcomes of AI. This problem is well illustrated by a study of judges collaborating with AI. Officially, the AI is supposed to make suggestions based on an analysis of case files, and the judge is to review those same case files to come to their own conclusion, which can then be compared with the computer’s outcome. However, Owing to the high time pressure judges’ face, they often decide to follow the AI’s recommendation without reviewing the case file separately.^29^ Furthermore, the hope that AI will lead to more time efficiency in health care is proving to be not as effective in practice. Anthropological research has shown that many professional caretakers actually end up with extra work after the introduction of technology.^30^ Although part of their work may be expedited thanks to collaboration with algorithms, this partnership also involves additional tasks that were not previously part of their role: filling out or reviewing certain digital forms and checking for false alarms. In addition, the health care sector, as is widely known, is currently overloaded in many places worldwide, and this is only expected to increase with rapid aging of the population. Can these overloaded employees be expected to collectively and continually train themselves in AI knowledge alongside their daily patient care responsibilities?

### Changing Skillsets

A fourth point that is currently relevant but will become even more crucial in the near future is the changing nature of skillsets. Current doctors and nurses gained extensive experience in using their own embodied experience and intuition for diagnosing and treating patients before they began working with algorithms. All professional caretakers are familiar with the “fingerspitzengefuhl” or gut feeling they rely on when making daily decisions related to patient care.^31^, ^32^, ^33^ Our analyses of in-depth interviews with professional caretakers in hospitals indicated multiple examples of the importance of intuition in clinical decision making: caretakers told how they often sense, or sometimes even smell, when something is amiss with a patient. They developed their sensory expertise not only in the workplace but also during their education where they learned to trust and use their vision, smell, and touch. However, the curriculum of these education programs is evolving as the workplace changes. In a 40-hour workweek where an increasing amount of hours shall be dedicated to familiarizing oneself with the technology that will be used, there remains less room for experience-based training on physical, intuitive diagnosis. This has consequences for the degree to which a human oversighter of AI systems is trained in the human skills that differentiate them from technology. In other words, Although an experienced, older physician may still be able to contrast their own intuitive diagnosis with the outcome of an AI, a younger physician may struggle with this. And how will they know if the AI is correct?^34^

Of course, education and curricula must keep up with the times. It is thus likely that the new generation of doctors and nurses will acquire new and crucial skills: they will undoubtedly be more digitally literate than most of their predecessors who did not learn these skills in school. Moreover, anthropologists found that nurses seem to develop a “mechanical sixth sense” after learning to work with new technologies, such as remote care via cameras: they become extra attentive to potential gaps in the system.^35^

However, they were able to develop this sense precisely because of the experience they gained from years of human interaction with patients, often through in-person visits. These visits taught them that a pile of dirty dishes that has been sitting in the kitchen for days or the unwashed hair of a patient, could be signals that more help is needed. After the introduction of remote care, nurses were concerned because they understood that they would have a harder time picking up on these signals through video calls, and for that reason, they developed additional tasks in their role, such as following up with patients or initiating contact and monitoring in other ways. This allowed them to continue delivering good care, but it was certainly not a time efficient process, and—more importantly for this point—it could not have been achieved without first gaining many human care skills.

### Conclusion

As of the utilization of ethical AI in health care will undoubtedly increase in the nearby future, it is crucial to consider not only the role of humans in decision making processes but also the challenges and limitations they face in this rapidly evolving landscape. This commentary highlighted a problem that was brought to light by dozens of doctors, e-health programmers, and other stakeholders in the context of an international study on the digitization of health care: namely, the pressure to quickly adapt to digital technologies and acquire computational skills can burden health care providers, potentially compromising patient care and ethical standards. The current focus on the human oversighter is, of course, crucial: without human oversight, AI systems may make mistakes that go unnoticed. However, the requirement for professional caretakers to become supervisors of AI systems often fails to fully address the substantial drawbacks associated with it, including the false sense of security it may provide in a context where health care providers are already under high work-pressure, and the unfair expectations placed on these providers to become digitally literate and, in the years to come, possibly even bear the individual responsibility that comes with it. This commentary mentioned 4 important issues: the growing expectations on explainable or transparent AI that lead to a façade of security, the lack of sufficient computational skills among many health care professionals, the increasing time constraints hindering their ability to undergo comprehensive training in programming or algorithms, and the diminishing human/intuitive skills. These challenges underscore the need for a more comprehensive approach to integrating AI into health care, one that considers not only the ethical implications but also the practical limitations and pressures faced by health care providers.

Moving forward, it is essential to address these challenges and work toward developing frameworks that support ethical AI implementation although safeguarding the well-being of both health care providers and patients. Only by tackling these issues with a realistic expectation of what human oversighters can, and cannot do, helps us to fully leverage the potential of AI to enhance health care outcomes while upholding ethical standards.

## Potential Competing Interests

Dr van Voorst reports that the Anthropology Department, University of Amsterdam, provided access to the digital scholarly library as a member of the research institution and facilitated a location for the workshops with clinicians who are discussed in the paper; reports starting grant from the European Research Committee.
